# Exploring the impact of remaining tooth structure and preparation size on the fracture resistance of endodontically treated mandibular premolars

**DOI:** 10.34172/joddd.025.42125

**Published:** 2025-03-31

**Authors:** Sıla Nur Usta, Hilal Tekkanat, Yiğitcan Sağlam, Cumhur Aydin

**Affiliations:** ^1^Department of Endodontics, Gulhane Faculty of Dentistry, University of Health Sciences, Ankara, Turkey

**Keywords:** Conservative instrumentation, Endodontics, Fracture resistance, Remaining dentin walls, Root canal preparation

## Abstract

**Background.:**

This study evaluated the influence of the combined effects of remaining dentin walls and mechanical instrumentation with taper sizes of 0.04 and 0.06 on the fracture resistance of endodontically treated mandibular premolars.

**Methods.:**

Seventy single-canal mandibular premolar teeth with similar dimensions were selected and divided into one control group and three main experimental groups: control group: intact teeth, group 1: four remaining walls, group 2: three remaining walls, and group 3: two remaining walls. Each group was further divided into two subgroups in terms of preparation size (0.06 or 0.04 taper). The teeth were restored with composite resin after creating restorative models and performing endodontic treatments. The fracture resistance of teeth was measured by the push-out test. Data were analyzed using two-way ANOVA and post hoc tests and the square of Spearman’s linear coefficient (*P*<0.05).

**Results.:**

The control group exhibited the highest fracture resistance compared to the experimental groups (*P*<0.05). Regardless of the taper size, group 3 showed the lowest fracture resistance values compared to groups 1 and 2 (*P*<0.05). Preparation sizes similarly affected the fracture strength of teeth (*P*>0.05). A significant positive association was found between the remaining dentin walls and fracture resistance (*P*<0.05).

**Conclusion.:**

This study emphasized the importance of preserving coronal dentin for fracture resistance in endodontically treated teeth (ETTs). Conservative instrumentation did not provide any advantages over traditional preparation in increasing fracture resistance.

## Introduction

 Root canal treatment aims to eliminate infected or necrotic pulp tissue and disinfect and seal the root canals to prevent future microbial contamination with a proper coronal restoration.^1^ Mechanical instrumentation plays a pivotal role in this procedure, utilizing a series of endodontic files to clean and shape the root canals and facilitate effective irrigation.^2^ However, this procedure can also weaken the root dentin structure of teeth, which might have already been compromised by caries and access cavity preparation, adversely affecting fracture resistance.^3,4^ Thus, a balanced approach is needed in selecting instrument sizes to ensure thorough root canal debridement while preserving as much dentin as possible to maintain structural integrity.

 Innovations in mechanical instrumentation using reduced tapers and/or apical sizes emphasize the importance of preserving dentin and maintaining natural tooth structure.^5^ While proper disinfection is the primary concern of conservative instrumentation, the literature presents divergent results on its effectiveness and impact.^6,7^ Additionally, the debate continues regarding the effect of larger taper sizes, which lead to the removal of excess dentin, negatively impacting fracture resistance of endodontically treated teeth (ETTs). Evidence remains inadequate to definitively link a specific preparation size to the fracture resistance. While some research indicates that larger taper sizes may reduce fracture resistance,^8^ this claim is still inconclusive and requires further validation through comprehensive studies.^9^ In this context, it is emphasized that an appropriate post-endodontic restoration that could enhance the biomechanical behavior by minimalizing the stress transmission to the root is essential.^10^

 The quantity of remaining dentinal walls in ETTs is another crucial factor influencing their resistance to fracture and, consequently, their longevity.^11^ In particular, endodontic access cavities and the loss of dentin walls due to the extent of caries can further reduce the fracture resistance of ETTs.^12^ Accordingly, a positive correlation has been demonstrated between the fracture resistance and the remaining sound dentinal walls.^13,14^ Therefore, the survival of ETTs is directly related to both the size of mechanical instrumentation and the amount of remaining coronal structure.^11^

 Although several studies have demonstrated the effects of taper size, cavity design, and remaining dentinal walls on the fracture resistance of ETTs,^15-17^ to the best of our knowledge, no study has evaluated the combined effects of taper size and the amount of remaining coronal structure on fracture resistance. Thus, this study assessed the influence of the remaining dentinal walls combined with mechanical instrumentation with taper sizes of 0.04 and 0.06 on the fracture resistance of ETTs. The null hypotheses tested were: (*i*) Fracture resistance of ETTs would not be affected by the remaining dentin walls; (*ii*) Fracture resistance of ETTs would not be affected by the preparation size.

## Methods

**Figure 1:**
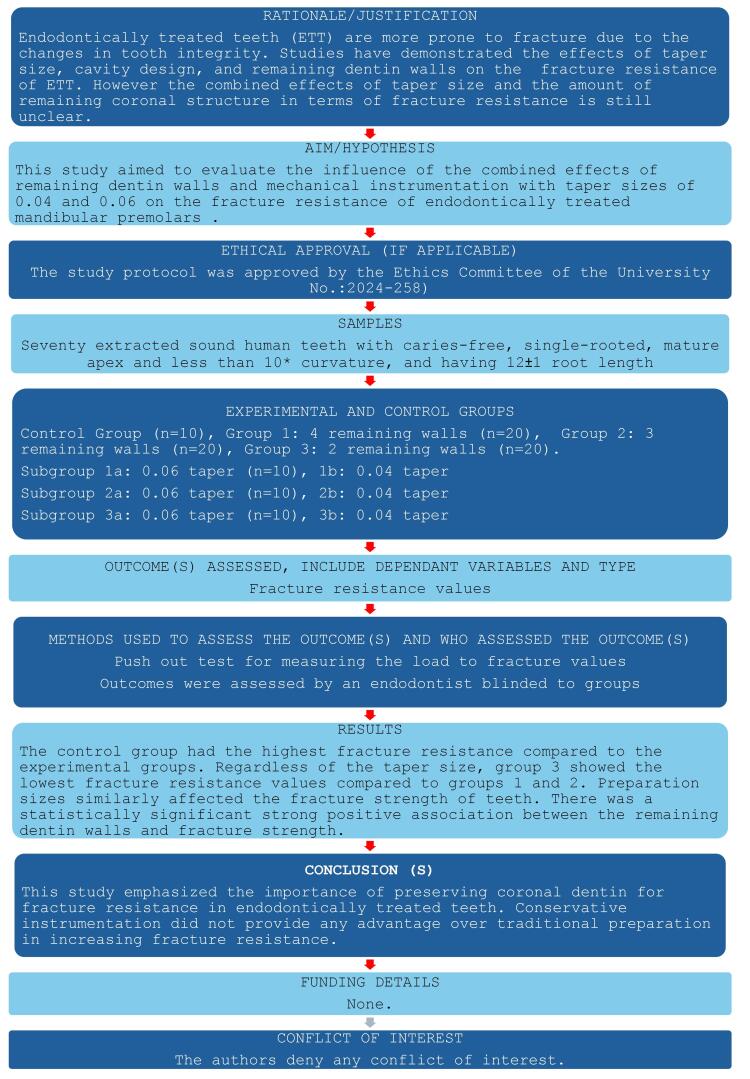
PRILE flow chart

 This laboratory study conformed to the Preferred Reporting Items for Laboratory Studies in Endodontology (PRILE) 2021 guidelines, as shown in Figure 1.^18^ The study protocol was approved by the Ethics Committee of the University (No.: 2024-258). The sample size was calculated based on a similar study in the literature^16^ with an effect size of 0.4921, type I error probability of 0.05, and a study power of 95%; consequently, the minimum required number of teeth per group was determined at n = 10.

 Seventy extracted sound, caries-free, single-rooted human teeth with mature apexes and less than 10º curvature,^19^ with 12 ± 1 mm of root length, were collected and evaluated under a stereomicroscope for any possible fractures or anatomical malformations. Buccolingual (BL) and mesiodistal (MD) dimensions of the selected teeth were measured using a digital caliper (Fideco, Shenzhen, China) to include teeth with similar dimensions. Teeth with calcified root canals or complex root canal anatomy, external or internal root resorption, and having apical constriction greater than a #10 K-file (Dentsply Maillefer, Ballaigues, Switzerland) were excluded. The periodontal tissues were then carefully removed from the external root surfaces of the selected teeth using periodontal curettes. Afterward, the teeth were stored in a 0.1% thymol solution at 4 °C for two months.

 In the experimental groups (n = 60), access cavities were prepared according to standard protocols, which involved completely removing the pulp chamber roof and establishing a direct, unobstructed pathway to the root canals.^20^ Ten intact teeth were used as a positive control group. The teeth were divided into 3 three main groups based on the number of remaining dentinal walls (n = 20 for each): group 1: four remaining walls, group 2: three remaining walls, and group 3: two remaining walls. Each group was further divided into two subgroups according to preparation size (0.06 or 0.04 taper) using randomization software (https://www.randomizer.org/). Each subgroup was presented as follows (n = 10):

1. Control group
2. Group 1a: four remaining walls + 0.06 taper
3. Group 2a: three remaining walls + 0.06 taper
4. Group 3a: two remaining walls + 0.06 taper
5. Group 1b: four remaining walls + 0.04 taper
6. Group 2b: three remaining walls + 0.04 taper
7. Group 3b: two remaining walls + 0.04 taper

Mesial and distal walls of the teeth were shaped using diamond burs (Ref.: 856L314-014, G&Z Instrumente, Lustenau, Austria), designed with rounded angles for the occlusal cavity. The occlusal widths of the preparations were set to two-thirds of the intercuspal distance. The proximal boxes were prepared with a width equal to half of the buccolingual dimensions of the teeth and an axial depth of 1.5 mm in the occlusogingival direction, as outlined in previous protocols.^21,22^ The depths were checked with a caliper and a periodontal probe (Hu-Friedy).

 Following the preparation of the restorative cavity models, the root canals of teeth in each group were prepared using a file system with 0.04 taper (EndoArt Smart Gold, Inci Dental, Istanbul, Turkey) or 0.06 taper (EndoArt Smart Gold, Inci Dental, Istanbul, Turkey), starting the tip size from #10 to #30. All the files were used in continuous rotation and positioned and activated at the root canal orifice. During endodontic treatments, 3 mL of 2.5% NaOCl (Microvem, Istanbul, Turkey) was delivered with a 30-G open-ended needle (Produits Dentaires SA, Vevey, Switzerland) attached to a 3-mL Luer-lock syringe between files. After instrumentation, the root canals were irrigated with 3 mL of 17% EDTA (Saver, Prime Dental Products, Maharashtra, India), followed by a final rinse with 3 mL of 2.5% NaOCl and distilled water. The root canals were then dried with sterile paper points (Diadent, Cheongju, Korea) and obturated with compatible gutta-percha using an epoxy resin-based root canal sealer with lateral condensation technique (Dia-Proseal, Diadent, Cheongju, Korea). Following the placement of Automatrix (Dentsply Sirona), the enamel and dentin of the access cavity were cleaned and etched using 37% phosphoric acid, with the enamel being treated for 30 seconds and the dentin for 15 seconds. Afterward, the cavity was rinsed for 30 seconds with a water/air syringe and gently air-dried to prevent desiccation. A light-cured primer bond adhesive (G Premio Bond; GC Corporation, Tokyo, Japan) was then applied, air-thinned gently, and cured with a light-emitting diode for 30 seconds. Following light-curing, the composite material (G-aenial Posterior; GC Corporation, Tokyo, Japan) was applied in horizontal layers, and each layer was light-cured for 20 seconds.

**Figure 2:**
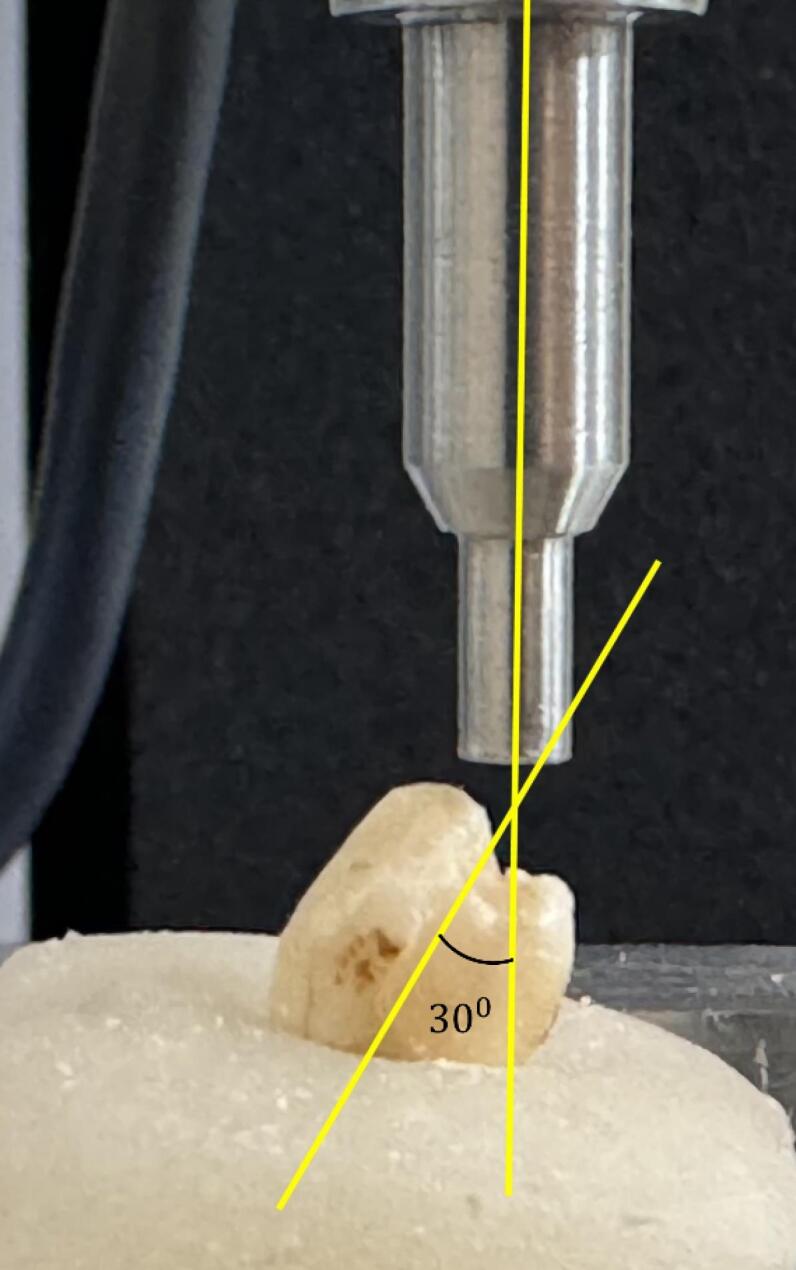
Experimental set-up of the fracture resistance test using the universal testing machine

 To assess the fracture resistance of ETT, the specimens were covered in a thin layer of wax up to 2 mm apical to the cementoenamel junction, simulating the periodontal ligament. Then, the teeth were embedded in acrylic molds and placed in a universal testing machine (AGS-X 10kN, Shimadzu Co., Kyoto, Japan). The teeth were continuously loaded in the central fossa at an angle of 30° from their long axis using a stainless steel cylindrical plunger of 3 mm (Figure 2).^16,17^ The push-out force was applied with a 1-mm/min speed coronoapically until a fracture occurred, and the load values were recorded in Newton (N).

###  Statistical analysis

 Data were analyzed using SPSS 26 (Chicago, IL, USA). The Kolmogorov-Smirnov and Levene tests were used to check the normal distribution and homogeneity of data, respectively. The statistical differences between the control and experimental groups were tested using one-way ANOVA and post hoc Tukey tests. Two-way ANOVA and post hoc Tukey tests were used to evaluate the cumulative effects of the remaining tooth structure and the preparation size on the fracture resistance of mandibular premolar teeth. The correlation between the remaining dentinal wall and resistance to fracture was evaluated using the square of Spearman’s linear coefficient. The level of significance was set at *P* < 0.05.

## Results

**Figure 3:**
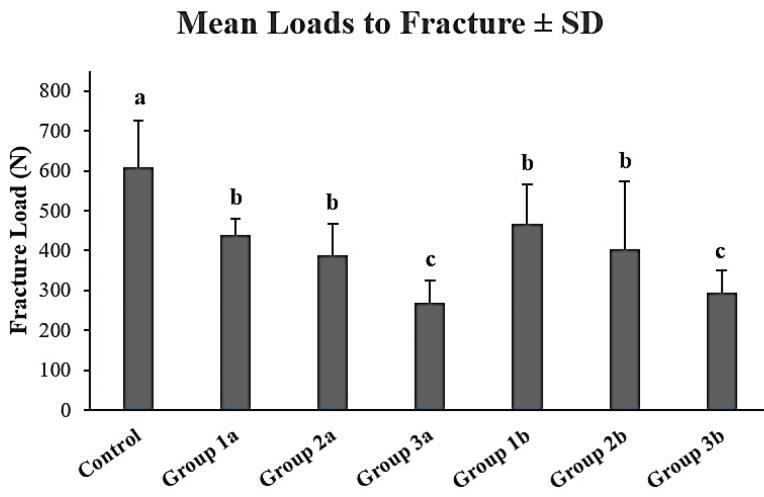
Mean ± standard deviations (SD) of the loads to fracture (N) in the experimental and control groups. Different superscript lowercase letters indicate a statistically significant difference (*P* < 0.05)

Figure 3 presents the mean fracture loads and standard deviations of the experimental and control groups. The control group exhibited the highest fracture resistance compared to the experimental groups (P < 0.05). Similar fracture loads were observed in groups 1a, 1b, and 2b (P > 0.05), which were significantly higher than those in groups 3a and 3b.

Table 1 demonstrates the combined effects of the remaining tooth structure and the preparation size on the fracture resistance of mandibular premolar teeth. While the fracture resistance was significantly affected by the remaining dentinal walls (*P* < 0.05), preparation sizes similarly affected the fracture resistance of ETTs (*P* > 0.05). Accordingly, regardless of the taper size, group 3 showed the lowest fracture resistance values compared to groups 1 and 2 (*P* < 0.05). Moreover, the results of the correlation analysis showed a significant positive association between the remaining dentinal walls and fracture resistance (correlation coefficient = 0.666, *P* < 0.001, R^2^: 0.309).

**Table 1:** Mean ± standard deviations (SD) of the load to fracture values of the groups in terms of taper size and remaining tooth wall

	**N**	**0.06**	**0.04**	**Comparison** ***P***** value**
Four remaining walls	20	439.84 ± 39.66^a,1^	466.99 ± 97.59^a,1^	0.000
Three remaining walls	20	388.16 ± 79.01^a,1^	403.75 ± 170.10^a,1^	
Two remaining walls	20	268.18 ± 55.31^b,1^	293.67 ± 57.20^b,1^	
Comparison *P* value		0.351		

Different superscript lowercase letters in the same column indicate a statistically significant difference (*P* < 0.05). The same superscript numbers in the same row indicate no statistically significant difference (*P* > 0.05).

## Discussion

 ETTs are more prone to fracture due to the changes in tooth integrity, dentin structure, and proprioception.^23^ Although the changes in tooth architecture are often attributed to access cavity preparation, root canal preparation is also an important step that may negatively affect the resistance of ETTs.^3^ Moreover, the quantity and integrity of the residual dentinal walls can influence the tooth’s ability to withstand occlusal forces and resist fracture after endodontic treatment.^11^ Therefore, this study aimed to assess the combined effects of remaining dentinal walls and preparation size on the fracture resistance of ETTs. Accordingly, while the first null hypothesis was rejected since the fracture strength was significantly affected by the remaining tooth structure, the second hypothesis was accepted.

 Standardization and storing conditions are critical in mechanical fracture testing in endodontics because they ensure the reliability, accuracy, and comparability of test results. Consistent sample preparation, testing environments, and loading protocols help minimize variability and allow for meaningful comparisons. In this sense, similar premolar teeth, through measurement of BL and MD dimensions and root lengths, were included in this study. Moreover, teeth were stored in wet conditions for two months to prevent dentin dehydration, which is a factor in decreasing fracture resistance.^24^

 In this study, different taper sizes of the same file system with identical metallurgical properties were used. This approach eliminated any potential differences that could arise from the structural characteristics of the file systems. In addition, all samples were restored with composite resin to mimic clinical conditions since it is commonly used to restore ETTs in routine dental practice.^25,26^

 The present study demonstrated that ETTs with more preserved coronal dentin exhibited significantly higher fracture resistance, regardless of the taper size used during instrumentation. Moreover, a strong positive correlation was observed between the remaining dentinal walls and fracture strength. These align with the study of Corsentino et al,^16^ who reported that the loss of mesial and distal walls reduced the fracture resistance of ETTs significantly. Moreover, Ibrahim et al^11^ also highlighted a positive linear relationship between the remaining coronal dentin surface area and fracture resistance. These findings can be explained by the fact that the largest losses in stiffness were related to the loss of marginal ridge integrity (63%), especially seen in teeth with two remaining walls.^27^ Nevertheless, it is also worth noting that ETTs were restored using a packable conventional composite resin to eliminate the potential fracture resistance-enhancing effect that might arise from the special properties of the restorative material in the scope of this study. In this context, indirect restoration with cusp replacement has been shown to be suitable for ETT restoration when a certain cavity extension is exceeded compared to direct restorations.^28,29^ Thus, employing varying post-endodontic restoration patterns might result in different outcomes.

 A recent systematic review could not reveal sufficient evidence regarding the effect of minimally invasive preparation on increasing fracture resistance of ETTs, primarily due to the inherent limitations of the studies and the moderate risk of bias. Thus, given the ongoing debate in the literature about the optimal preparation size, divergent results have been found based on the different methodologies. Accordingly, while some studies have suggested that larger taper sizes may weaken the tooth by removing excessive dentin,^3,30,31^ others have shown that the fracture resistance was unaffected by preparation size.^17,32,33^ The present study’s results suggest that taper size alone is not a decisive factor when a sufficient amount of dentin is preserved. This could be observed due to the effect of the coronal restoration on the fracture resistance of ETTs since restored teeth could regain up to 72% of their fracture resistance compared to unrestored teeth.^10^ Therefore, it was considered that a suitable coronal restoration performed following endodontic treatment could minimize the effect of the preparation size on ETTs. However, this study only explored the effects of two specific taper sizes. A broader range of taper sizes and their long-term impact on the fracture resistance of ETTs need to be examined to inform clinical decision-making.

 It is important to indicate that although the findings of this study are robust, they are derived from in vitro conditions, which may not fully replicate the complex biomechanical environment of the oral cavity. Therefore, while the results provide valuable insights, they should be interpreted cautiously in clinical practice. Other researchers have also acknowledged this limitation and emphasized the need for clinical studies to validate laboratory findings.

## Conclusion

 This study underscores the paramount importance of preserving coronal dentin to maintain fracture resistance of ETTs. Conservative preparation techniques did not improve the strength of ETTs compared to traditional preparation. Further research is necessary to explore the interplay between various endodontic techniques and their impact on tooth strength to optimize treatment outcomes and improve longevity.

## Competing Interests

 The authors deny any conflicts of interest.

## Ethical Approval

 The study protocol was approved by the Ethics Committee of the University of Health Sciences (No.: 2024-258).
